# Characterizing the Input-Output Function of the Olfactory-Limbic Pathway in the Guinea Pig

**DOI:** 10.1155/2015/359590

**Published:** 2015-07-28

**Authors:** Gian Luca Breschi, Carlo Ciliberto, Thierry Nieus, Lorenzo Rosasco, Stefano Taverna, Michela Chiappalone, Valentina Pasquale

**Affiliations:** ^1^Department of Neuroscience and Brain Technologies, Istituto Italiano di Tecnologia, Via Morego 30, 16163 Genova, Italy; ^2^Laboratory for Computational and Statistical Learning, Istituto Italiano di Tecnologia, Via Morego 30, 16163 Genova, Italy; ^3^Department of Informatics, Bioengineering, Robotics and System Engineering (DIBRIS), Università degli studi di Genova, Via Dodecaneso 35, 16146 Genova, Italy

## Abstract

Nowadays the neuroscientific community is taking more and more advantage of the continuous interaction between engineers and computational neuroscientists in order to develop neuroprostheses aimed at replacing damaged brain areas with artificial devices. To this end, a technological effort is required to develop neural network models which can be fed with the recorded electrophysiological patterns to yield the correct brain stimulation to recover the desired functions. In this paper we present a machine learning approach to derive the input-output function of the olfactory-limbic pathway in the *in vitro* whole brain of guinea pig, less complex and more controllable than an *in vivo* system. We first experimentally characterized the neuronal pathway by delivering different sets of electrical stimuli from the lateral olfactory tract (LOT) and by recording the corresponding responses in the lateral entorhinal cortex (l-ERC). As a second step, we used information theory to evaluate how much information output features carry about the input. Finally we used the acquired data to learn the LOT-l-ERC “I/O function,” by means of the kernel regularized least squares method, able to predict l-ERC responses on the basis of LOT stimulation features. Our modeling approach can be further exploited for brain prostheses applications.

## 1. Introduction

Thanks to recent advances in neurotechnology and neurosurgery the possibility of implanting smart devices in the brain to replace damaged neuronal circuits or deliver appropriate electrical stimulation is opening the way to innovative treatment of neurological disorders, from epilepsy to stroke [1–4]. Proposed solutions range from simple activity-dependent stimulators [3, 4], which monitor the activity of selected brain regions and deliver electrical pulses to other regions depending on the detected patterns, to more complex modeling approaches [1, 2], which aim at learning the input/output (I/O) function of specific neuronal circuits to be replaced or “repaired.” In both approaches electrical stimulation is continuously modulated in response to specific recorded patterns of activity, in real time and in a closed-loop fashion.

In this context, machine learning techniques, which can operate either simple pattern recognition or more complex statistical modeling, can be of great help in abstracting the I/O behavior of neuronal circuits. For instance, it has been suggested that some brain regions, such as the dentate gyrus of the hippocampus, may provide the proper neuronal substrate to generate a sparse representation of its input [5]. Such representation is at the basis of many machine learning tools that, as the DG does, can perform “pattern separation” tasks [6].

In this work we have made use of machine learning techniques to model the behavior of the olfactory-limbic pathway in the isolated whole brain (IWB) of the guinea pig. The IWB represents an experimental approach combining the advantages of both* in vitro* (accessibility, controllability) and* in vivo* (structural integrity) conditions [7], thus providing an ideal biological substrate for this kind of studies. Electrical stimulation of the lateral olfactory tract (LOT) reliably activates mono- and polysynaptic responses in the olfactory cortices and limbic structures [8, 9]. Therefore, these cortical regions can be investigated, from single neuron up to neuronal network level, using intracellular, multiunit, and field potential recordings.

By using different paired-pulse stimulation protocols we first characterized the I/O function of the lateral entorhinal cortex (l-ERC) upon LOT stimulation. Then, we employed kernel regularized least squares (KRLS) approaches [10] to approximate the transfer functions governing the causal relationship between LOT electrical stimulation and l-ERC response in terms of number of evoked spikes and 1st spike latency. Our results show that we were able to fit these functions with good approximation, especially for the number of evoked spikes.

These results might have a possible application in the framework of the design and development of innovative neuroprostheses and demonstrate that machine learning techniques can be successfully applied to model the statistical relationship between inputs and neural outputs.

## 2. Materials and Methods

### 2.1. *In Vitro* Isolated Brain of Guinea Pig: Preparation and Experimental Setup

We performed electrophysiological recordings on the guinea pig isolated whole brain (IWB) maintained* in vitro* by perfusing a complex saline solution throughout the brain vascular system. The peculiarity of this experimental model is the complete preservation of the neuronal and vascular structure integrity; therefore, the isolated guinea pig brain is ideal for studying local and long-range neuronal interactions in unrestricted brain networks.

**Figure 1:**
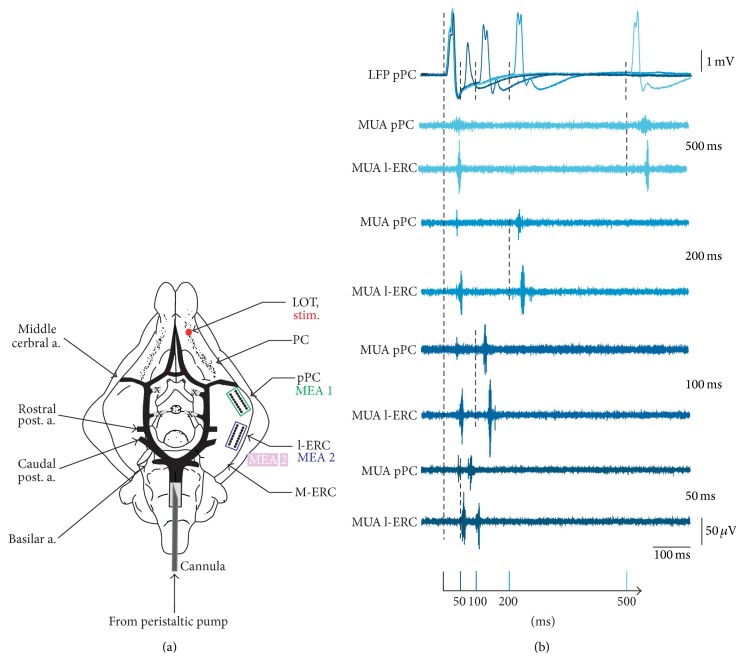
Experimental configuration and raw data. (a) The left drawing depicts the ventral surface of the IWB with the principal vessel highlighted in black and the two cortical areas (pPC and l-ERC). The positions of two schematized MEAs in pPC and l-ERC (in green and violet, resp.) and of the stimulant electrode (S1 in the LOT) are shown. (b) Example of the recordings obtained during the application of an IPI variation protocol. The evoked field potentials (LFP, top traces) observed in the pPC are evoked from the LOT. The corresponding MUA traces, shown in the lower panels, are simultaneously recorded in the pPC and l-ERC.

Young adult Hartley guinea pigs (150–300 g, Charles River) were anesthetized with sodium thiopental (125 mg/kg, i.p.) and transcardially perfused with a cold (4 C), oxygenated (95% O_2_, 5% CO_2_) saline solution composed of 126 mM NaCl, 3 mM KCl, 1.2 mM KH_2_PO_4_, 1.3 mM MgSO_4_, 2.4 mM CaCl_2_, 26 mM NaHCO_3_, 15 mM glucose, 2.1 mM HEPES, and 3% dextran (MW 70,000). The pH of the solution was corrected to 7.1 with HCl. The brain was dissected out, transferred to a recording chamber, and perfused at 7 mL/min with the above solution (pH = 7.3, 15 C) via a peristaltic pump (Minipulse II, Gilson, France) through a cannula inserted in the basilar artery (see Figure 1(a)). Prior to recording, the temperature of the preparation was gradually increased to 32°C (0.2 C/min) [11–13].

We recorded evoked local field potentials (LFP) and multiunit activity (MUA) simultaneously in posterior piriform cortex (pPC) and lateral entorhinal cortex (l-ERC). LFP responses were evoked by electrical stimulation (0.07–0.3 mA, 0.36 ms) of the LOT using a custom-made bipolar electrode made of twisted, insulated silver wires (S1 in Figure 1(a)).

LFP and MUA were recorded using two microwire arrays (Tucker-Davis Technologies, Alachua, FL, USA) featuring 16 tungsten planar recording wires (filament diameter 50 *μ*m, tip angle 45°), each separated by 250 *μ*m (impedance 30–40 KΩ). The electrodes were implanted in the superficial layers of the pPC and l-ERC (200–500 *μ*m from pial surface). The extracellular signals were acquired using a PBX3 preamplifier (Plexon, Dallas, TX, USA) configured to separately process spikes (bandwidth range: 150 Hz–8 KHz) and local field potentials (0.7–300 Hz). Data were digitized at 10 KHz using a PCI-6071E A/D board (National Instruments, Austin, TX, USA) and stored on the hard drive of a computer. Recordings were performed using ELPHO software developed by Dr. Vadym Gnatkovsky at the C. Besta Neurological Institute (Milan, Italy).

Stimulation of the LOT activated two associative potentials (i.e., the mono- and disynaptic components of the evoked response) in the pPC, while after 3–5 ms (a time lag attributable to a monosynaptic activation) a synaptic response was detected in the l-ERC.

### 2.2. Experimental Protocols

We applied different electrical stimulation protocols to the LOT. In the first paradigm (*n* = 6) we modified the interpulse interval (IPI) of a paired pulse: 50, 100, 200, and 500 ms. Seventy-five percent of the stimulus intensity necessary to evoke a complete and stable LFP response was used throughout the experiment. In a second stimulation protocol (*n* = 6) we progressively increased the intensity of a paired pulse (200 ms IPI), from the minimal to the maximal amplitude necessary to elicit an evoked response. In both cases the paired pulses were delivered at 0.2 Hz.

### 2.3. Data Preprocessing

Raw data acquired by the ELPHO software were loaded into MATLAB (the Mathworks Inc., Natick, MA, USA) for offline processing. First, raw traces were bandpass filtered (by applying a second-order bandpass elliptic filter, bandwidth 800 Hz–3 kHz) to select spiking activity. Stimulation artifacts were suppressed using an offline MATLAB implementation of the SALPA (Subtraction of Artifacts by Local Polynomial Approximation) algorithm [14]. Highly noisy channels were visually excluded from the analysis. Then, filtered raw data were spike detected by means of the Precise Timing Spike Detection (PTSD) algorithm [15] (peak lifetime period = 2 ms; refractory period = 1 ms; threshold = ±9 times the estimated noise standard deviation). The result of the spike detection procedure consists of a series of point processes (i.e., spike trains), one for each recording channel [16]. We evaluated the evoked response by computing the Peristimulus Time Histogram (PSTH) [17] for each recording channel of the array and for the full array (time bin = 2 ms). The considered time window was between −10 ms and twice the IPI relative to the 1st pulse stimulus onset.

The strength (or intensity) of each response to electrical stimulation was evaluated by computing the average number of evoked spikes on each recording channel (on a time window equal to the IPI). Instead, the latency of the response was evaluated as the average delay of the first evoked spike with respect to the stimulus onset (either 1st or 2nd pulse). The response strength can be computed upon the application either of the 1st pulse or of the 2nd pulse, and the two measures can be summed to compute the global response strength (i.e., total number of evoked spikes).

As far as the strength is concerned, in order to merge results of different experiments we operated a normalization procedure. For the stimulation intensity variation experiments, we divided all strengths by the global average strength obtained for the maximal stimulation intensity. For the IPI variation experiments, we divided all strengths by the global average strength obtained for an IPI equal to 500 ms over all electrodes. On the contrary, latencies were not subject to any normalization. For the stimulation intensity variation protocol, since the actual values of stimulation currents vary in different experiments, in each stimulation session we normalized all stimulation intensity values to the maximal one, which has been chosen as the lowest current value evoking a stable response (by looking at the LFP signal). In this way, different experiments and stimulation sessions can be compared and considered altogether.

### 2.4. Mutual Information Computation

We performed mutual information measurements to determine the discriminative power of each single feature obtained from the experimental data. The mutual information [18] is given by MI(*R*; *S*) = *H*(*R*) − *H*(*R*∣*S*), where *H*(*R*) represents the entropy of the responses and *H*(*R*∣*S*) is the entropy of the responses conditioned to the stimulus. The latter term can be regarded as the uncertainty of the association between a stimulus and its responses. The highest the uncertainty *H*(*R*∣*S*) is, the less the mutual information and the corresponding discriminative power of the features will be. In particular, to compare the relevance of the features across different experiments we considered the coding fraction: cf(*R*; *S*) = MI(*R*; *S*)/*H*(*R*). For this analysis, we focused on experiments where we varied the input IPI (ranging from 50 to 500 ms) and the stimulus amplitude (ranging from 50 to 130 *μ*A).

### 2.5. Machine Learning Methods to Learn and Predict Output Features from LOT Stimuli

To predict the output responses (# spikes or latency) elicited by a given LOT stimulation, we adopted a supervised nonparametric machine learning approach: kernel regularized least squares (KRLS). The method works as follows: given a training set of *n* input observations *x*_1_,…, *x*_*n*_ (in our case the IPI and stimulus amplitude values) and corresponding outputs *y*_1_,…, *y*_*n*_ (the # spikes/latency), the system learns the function *f*(*x*) = *y* that produced such observations. In nonparametric settings, the function *f* is modeled as the linear combination *f*(*x*) = ∑_*i*=1_^*k*^ *a*_*i*_ *φ*_*i*_(*x*) of multiple “atoms” or basis functions *φ*_1_,…, *φ*_*k*_ that can account for different nonlinearities in the system.

More precisely, KRLS, which can be seen as a nonparametric extension of ridge regression, finds a function as superposition of “kernels” centered at the training set points (see below). Similar to more basic least squares methods, the coefficients of the expansion can be found by solving a linear system which is now defined by the kernel. By choosing different kernels, the method allows modeling different kinds of nonlinear functional dependencies, while avoiding overfitting, that is, finding statistically stable models.

In this work we considered two possible choices of basis functions, namely, linear and Radial Basis Function (RBF). The linear approach is used as a baseline and consists in simply fitting a linear model on the training data. The RBF approach on the other hand consists in using a set of basis functions *φ*_*i*_(*x*) = exp⁡(−‖*x* − *x*_*i*_‖^2^/*σ*) each centered on a training input *x*_*i*_ and learning the corresponding coefficients *a*_1_,…, *a*_*n*_ that characterize the best fitting function *f*(*x*) = ∑_*i*=1_^*n*^ *a*_*i*_ *φ*_*i*_(*x*). To solve this problem we employed the MATLAB implementation of KRLS in the custom toolbox GURLS [19] developed by the Laboratory for Computational and Statistical Learning (LCSL) at the Massachusetts Institute of Technology (MIT). The learning model is characterized by two main hyperparameters, namely, the regularization, *λ*, and the Gaussian bandwidth of the RBF atoms, *σ*. We selected the best hyperparameters according to the cross-validation procedure described below.

### 2.6. Accuracy Computation and Model Validation

We evaluated the predictive capabilities of the learning systems trained on the collected dataset according to different measures of performance. Specifically, for the problem of predicting the number of spikes elicited by a given LOT stimulus we reported the classification accuracy, namely, the ratio of correctly predicted number of spikes with respect to the total number of predictions made. For the latency estimation problem we used the normalized mean squared error (nMSE) which reports the squared error (*f*(*x*) − *y*)^2^ normalized with respect to the variance of the output Var⁡(*y*). According to this measure, a nMSE of value 1.0 would correspond to a system unable to infer any relation between the inputs *x* and the outputs *y* while a value of nMSE = 0 would correspond to a system that is perfectly reconstructing the output observation from the input stimulus.

Model selection and validation of the learning systems described above were performed following a 10-fold cross-validation procedure: we subdivided the whole dataset acquired during our experiments in 10 distinct subparts. We used 9 out of 10 of these smaller datasets to train the KRLS learning system as described above and the remaining dataset to evaluate its prediction performance according to the corresponding measure (accuracy for # spikes, nMSE for latency estimation). We accounted for statistical variability by averaging the prediction results over multiple runs of this train/test protocol, one for each of the 10 cross-validation datasets.

### 2.7. Statistical Analysis

Whenever the normality assumption failed (checked by applying Kolmogorov-Smirnov test, *P* level 0.01), nonparametric statistical tests were applied (e.g., Wilcoxon rank sum test, Kruskal-Wallis ANOVA). Unless differently specified, all data are reported as mean ± standard error of the mean. In box plots, the median value and 25th–75th percentiles are indicated by the box, whereas whiskers indicate either 95th (upper) or 5th percentile (lower). All statistical analyses have been performed by using OriginPro v 8.6 (OriginLab Corporation, Northampton, MA 01060, USA) or MATLAB.

## 3. Results and Discussion

In order to investigate the input/output (I/O) function of the olfactory-limbic pathway of the* in vitro* guinea pig brain, we first performed electrophysiological recordings of the pPC and l-ERC by using MEAs upon stimulation of the LOT. Such stimulation activated associative potentials throughout the olfactory cortex. These potentials were characterized by two distinct peaks: the mono- and disynaptic response (see LFP traces in Figure 1(b)). The former corresponds to the activation of apical dendrites in the superficial layer of the pPC, while the latter is sustained by intra-pPC fibers and by associative fibers originating from neighboring cortical structures. l-ERC responses were characterized by a large wave component that represents the direct propagation of the olfactory input, followed by large-amplitude polysynaptic response [8, 20, 21].

Upon LOT stimulation, polysynaptic response of pPC showed latency of 36.64 ± 3.62 ms, whereas the l-ERC, which represents the output of the pPC, was activated after 41.04 ± 1.51 ms.

### 3.1. Electrophysiological Characterization of the I/O Behavior of the LOT-l-ERC Pathway

**Figure 2:**
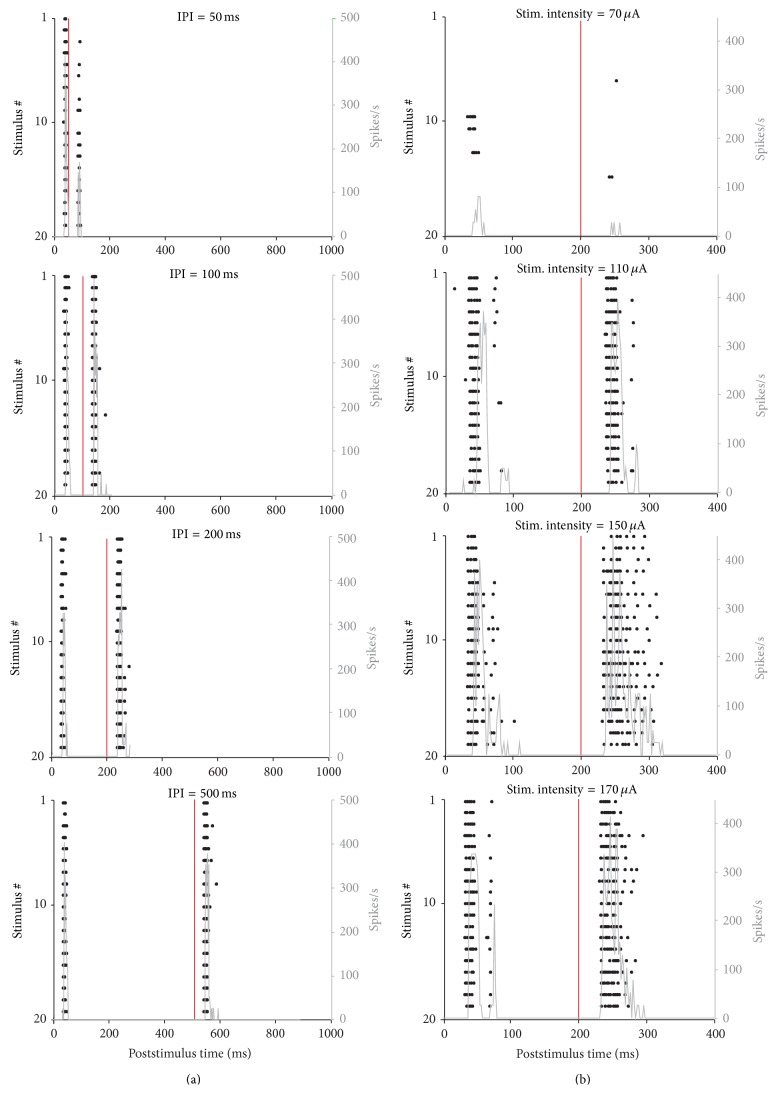
Raster plots and PSTH functions of selected electrodes for different tested values of IPI (a) and stimulus intensity (b). The stimulation raster plots show the spiking activity recorded by a single electrode in response to all stimulation trials. The time window is 1 s; 20 stimulation trials have been shown for each condition.

Figure 2 shows representative examples of the spiking activity recorded by one electrode of the MEA in l-ERC during typical stimulation sessions. In Figure 2(a) we varied the IPI between the two pulses from 50 to 500 ms, whereas in Figure 2(b) we varied the stimulation intensity from 70 to 170 *μ*A. We plotted the stimulation raster plots (in black), where each row represents the spike train evoked by a single instance of a paired-pulse stimulus, and then superimposed the corresponding PSTH (light grey). Responses were quite reproducible for different stimulation trials, and PSTHs show sharp peaks at precise latencies. By varying the IPI, the response to the second pulse appears to be facilitated for IPI = 100 ms and IPI = 200 ms (Figure 2(a)). While increasing the stimulation intensity, responses to both pulses increase, but the effect is much more prominent for responses to the 2nd pulse (Figure 2(b)).

As described in Section 2, we considered different output features, that is, the average number of spikes evoked by either the 1st or the 2nd pulse, the total number of evoked spikes (by both pulses), and the average latency of the 1st evoked spike by either the 1st or the 2nd pulse. We measured these features for each electrode of the MEA implanted in l-ERC and for each tested value of the input.

**Figure 3:**
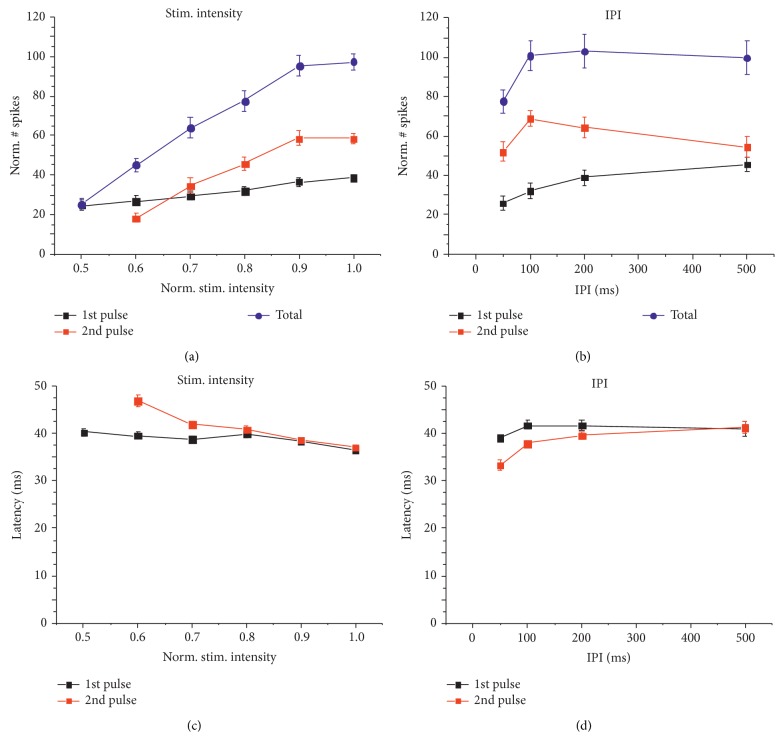
Average response curves for the stimulation intensity and the IPI variation protocols. (a, b) Normalized number of spikes in l-ERC upon LOT stimulation, when varying the normalized stimulation intensity (a) or the IPI (b). The normalized number of spikes is reported as a percentage of the reference response, which is the average total number of spikes obtained for the maximal stimulation intensity. Black curves report responses evoked by the 1st and red curves responses by the 2nd pulse. Blue curves report the total number of spikes. (c, d) The latency of the response is depicted as the average delay of the first evoked spike with respect to the stimulus onset, obtained by varying either the stimulation intensity (c) or the IPI (d). All graphs report mean ± SEM.

In Figure 3, we reported the mean ± standard error of the mean (SEM) of output features as a function of input parameters. As a confirmation of the qualitative observations made for Figure 2, we observed paired-pulse facilitation for intensities ranging from 70% to 100% of the maximal amplitude (Figure 3(a)). An additional increase did not induce any further spike increment (data not shown). Interestingly, the spike latency differences between the first and the second stimuli were abolished as the PP facilitation increased (Figure 3(c)).

In l-ERC, a paired-pulse facilitation mechanism is activated by IPI higher than 50 ms and reaches its maximum for IPI = 100 ms (Figure 3(b)). Hence, the number of spikes which correlated with the disynaptic component of the evoked field potential (see also Figure 1) increased in accordance with the IPI (Figure 3(c)). The conditioning effect of the first stimuli started to decline for IPI higher than 100 ms. In fact, for an IPI of 500 ms the two responses were independent as the number of evoked spikes is almost equal. Furthermore, the facilitation itself likely explains the significantly shorter latencies at the second pulse for IPIs shorter than 200 ms (Figure 3(d)).

### 3.2. Modeling Approach

We focused on the modeling of the direct olfactory pathway in order to derive a function allowing us to predict the l-ERC electrophysiological activity from the features of the LOT stimulation.

**Figure 4:**
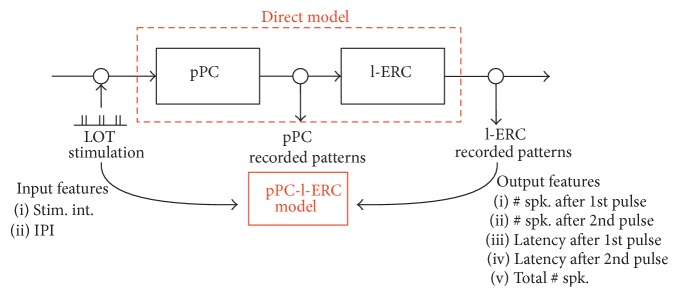
pPC-l-ERC model diagram. The “pPC-l-ERC model” is estimated from the experiments of LOT stimulation and l-ERC multichannel recording. The input features that we varied during the experiments are the stimulus intensity (stim. int., cf. protocol 1) and the interpulse interval (IPI, cf. protocol 2), whereas the output features are the number of evoked spikes after 1st and 2nd pulse, the total number of spikes, and the latency of the first evoked spike after 1st and 2nd pulse.

In Figure 4 we reported a graphical representation of the “pPC-l-ERC model.” Our goal is to build a system that is able to accurately replicate the neural response of the l-ERC to a given LOT stimulation in terms of output features (Figure 4). To this end we used a set of empirical measurements obtained according to the stimulation protocols described above in order to train a prediction system.

As already anticipated, to characterize the neural activity evoked by a given stimulus, we considered two distinct sets of output features measured by the l-ERC MEA, one related to the number of spikes elicited by each stimulus, the other recording the latency of such response (Figure 4).

### 3.3. Mutual Information between Inputs and Outputs

**Figure 5:**
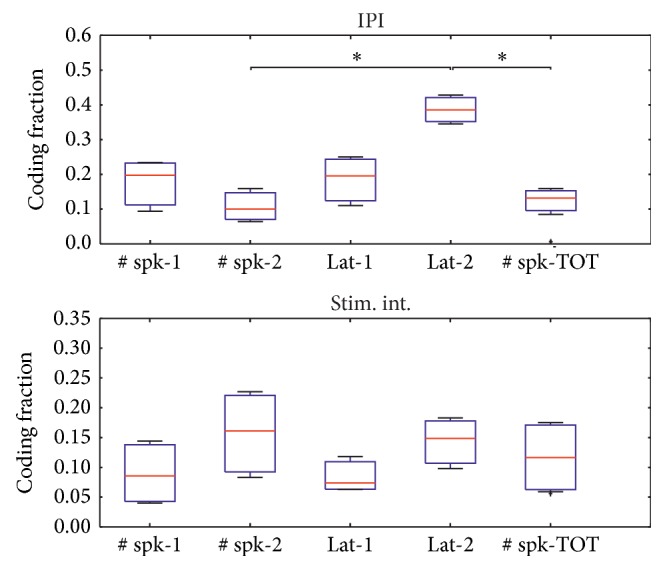
Mutual information analysis between inputs and outputs. The coding fraction (see text) was quantified for all output features (# spk-1: number of spikes after 1st pulse; # spk-2: number of spikes after 2nd pulse; lat-1: latency after 1st pulse; lat-2: latency after 2nd pulse; # spk-TOT: total number of evoked spikes) analyzed in the IPI (*n* = 6) and stimulus intensity (stim. int.) (*n* = 6) variation experiments. Box plots report the distributions of coding fraction for all experiments. *∗* denotes *P* < 0.05 significance level (Kruskal-Wallis ANOVA for ranks, with Bonferroni correction).

The mutual information analysis showed that for the IPI experiments the latency of the first spike after the second stimulus was the most significant (Figure 5—top panel) among the tested features. This is of particular relevance because the temporal encoding of stimuli is reflected into the responses. This analysis also shows that the latency is a much more effective feature than the number of spikes evoked by the second stimulus. For the stimulus intensity experiments the latency of the first spike after the second stimulus was still informative but also the number of spikes evoked by the second stimulus was a relevant feature for the discriminative task. Interestingly the number of spikes after 2nd pulse was consistently, over all data sets, more informative than number of spikes after 1st pulse, suggesting that paired stimuli better reflect the different stimulation amplitude adopted in the experiments.

### 3.4. Output Prediction from LOT Stimuli

**Figure 6:**
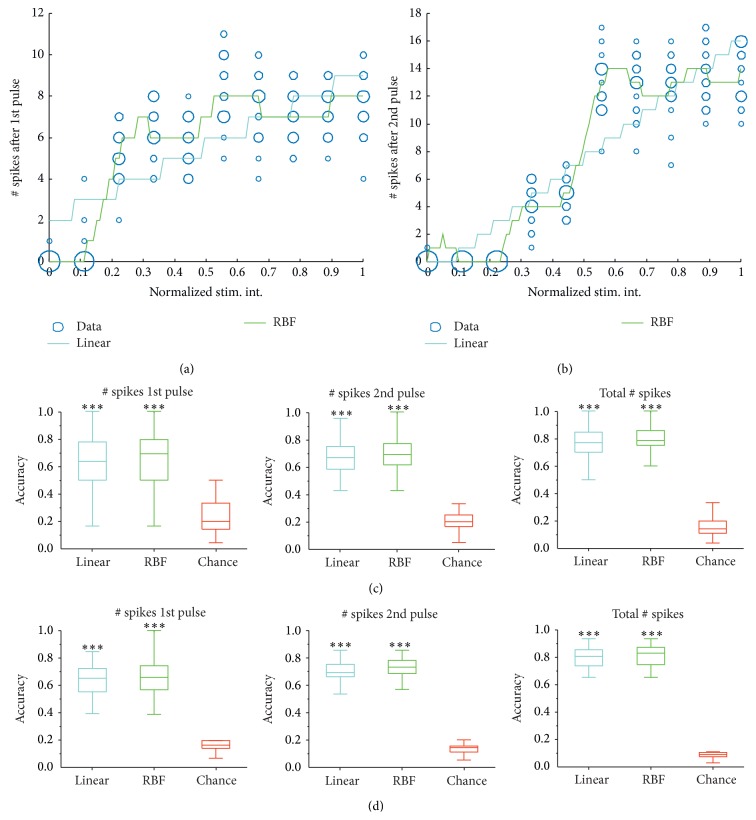
Results of neural activity prediction for the KRLS algorithm with linear (cyan) and RBF (green) kernels. (a, b) # spikes recorded by a representative electrode as the LOT normalized stimulus intensity varied from 0 to 1. Blue circles report the frequency with which a specific number of spikes were recorded for a given stimulus intensity (e.g., larger circles correspond to more frequent # spikes). (a) # spikes after the 1st pulse. (b) # spikes after 2nd pulse. (c, d) Distributions of prediction accuracies reported in box plot format (all recording channels, all experiments). Linear (cyan) and RBF (green) approaches are compared with chance levels (red). *∗∗∗* denotes *P* < 0.001 significance level with respect to chance levels (Kruskal-Wallis ANOVA for ranks, with Bonferroni correction). (c) Accuracy distribution for # spikes after 1st pulse (left), # spikes after the 2nd pulse (middle), and total # spikes (right) for IPI variation. (d) Accuracy distribution for # spikes after 1st pulse (left) # spikes after the 2nd pulse (middle), and total # spikes (right) for stimulus intensity (stim. int.) variation.

In Figures 6(a) and 6(b) we reported the number of spikes after 1st (Figure 6(a)) and 2nd pulse (Figure 6(b)) predicted by a trained KRLS system using an RBF (Gaussian, in green) and a linear kernel (in cyan) for a representative electrode as the LOT normalized stimulus amplitude varies from 0 to 1. Blue circles report the frequency of a fixed number of spikes observed for a given LOT stimulus. The radius of the circle is directly proportional to such value (e.g., spikes that are more frequently observed/most likely to be recorded are associated with larger circles). From a qualitative perspective it can be noticed that the nonlinearity introduced by the Gaussian kernel is extremely helpful to fit the data and indeed almost always predicts the numbers of spikes that are associated with most frequent classes. This is not true for the KRLS predictor using linear kernel; see, for instance, the prediction produced for inputs between 0.5 and 0.6 LOT stimulus amplitude in Figure 6(b).

To quantitatively measure the generalization performance of the two machine learning approaches described in Section 2 we reported—as a box plot—the accuracy (in predicting the correct number of spikes) measured independently for each electrode channel (among the 16 available). We invalidated those electrode channels for which less than 10 observations were available. As a baseline measure we also reported the chance levels, that is, the probability of predicting the correct number of spikes by randomly choosing such value among those available in training. For instance, if the maximum number of spikes recorded during training was 12, the chance of correct guessing will be 1/12.

From these experiments it can be clearly noticed that the stimulus amplitude (Figure 6(c)) is strongly correlated to the number of neural spikes evoked. Indeed, for both linear and nonlinear fitting approaches, the observed accuracy is relatively concentrated around high accuracy values (65–70%) with respect to chance (around 10%). On the contrary, the IPI value should be unrelated to the number of spikes elicited by the first stimulus and, indeed, as it can be noticed from Figure 6(d), the prediction accuracies of both linear and RBF approaches have a large variance. As expected, the IPI is strongly related to the number of spikes elicited by the second stimulus, leading again to prediction accuracies that concentrate around high values.

**Figure 7:**
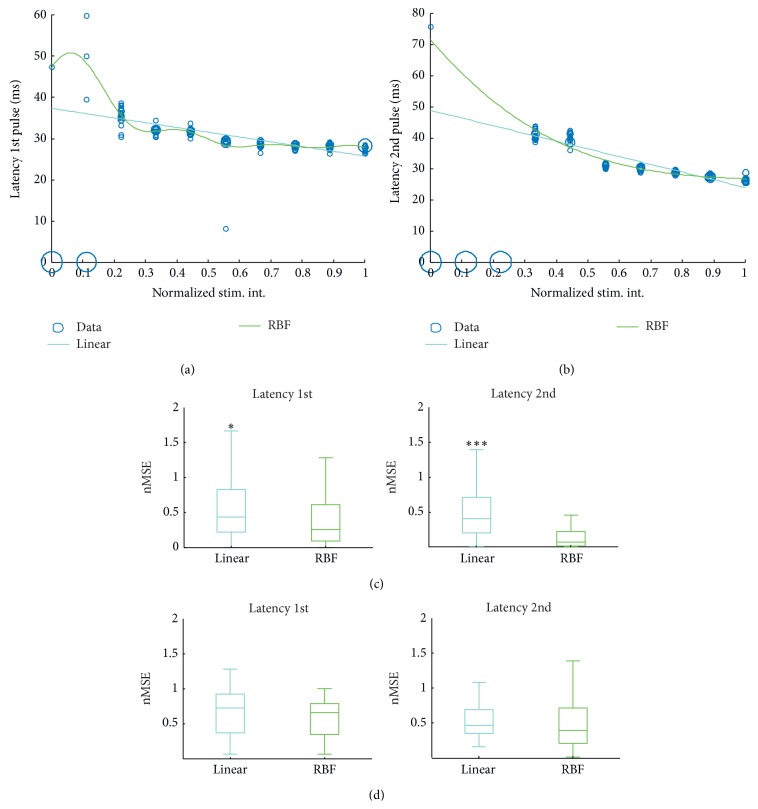
Results of prediction of the latency between LOT stimulus and neural response. (a, b) Observed and predicted latency values of the neural activity elicited by LOT stimuli. Blue circles correspond to observed latencies. Predictions provided by KRLS algorithm trained on previous empirical evidence are reported: cyan for linear kernel and green for Gaussian (RBF) kernel. (a) Latency after 1st pulse. (b) Latency after 2nd pulse. (c, d) Distributions of the normalized mean squared error (Nmse; see text for a definition) reported in box plot format. *∗∗∗* and *∗* denote, respectively, *P* < 0.001 and *P* < 0.05 significance levels (Wilcoxon rank sum test). (c) nMSE distributions of the predicted latency after 1st pulse (left) and 2nd pulse (right) in the experiments of IPI variation. (d) nMSE distributions of the predicted latency after 1st pulse (left) and 2nd pulse (right) in the experiments of stimulus intensity (stim. int.) variation.

The time incurring between a LOT stimulus and the neural activity it induces is also a feature in which we are interested. This latency is measured in milliseconds (ms) for each channel of the MEA in the l-ERC and can be modeled as a one-dimensional process that directly depends on the LOT stimulus input. In analogy to the experiments performed to estimate the correlation between IPI/stimulus amplitude and the number of elicited spikes, we report in Figures 7(a) and 7(b) the performance of both linear and RBF methods when predicting the latency of neural response after the first and second stimulus. Similar to the previous experiments, the blue dots represent the observed behavior of the system during the stimulation process, with responses clustered around the most likely latency values. As can be noticed from the plot, the linear method leads to more robust but less accurate estimation. Instead, the nonlinear approach with the Gaussian kernel is more sensitive to small variations but replicates more accurately the true “pPC → l-ERC” behavior.

As already mentioned in Section 2, we measured prediction performance of the machine learning system approaches employed in this work in terms of their normalized mean squared error (nMSE). Analogously to the previous analysis on the number of spikes, we performed the latency prediction experiments for each of the 16 electrode channels. We report the nMSE values measured as a box plot in Figures 7(c) and 7(d).

Notice that the spike latency observed after the first pulse is logically independent of the IPI value; therefore, the statistical analysis boils down to the estimation of the expectation of the spike latency based on all observations regardless of their IPI value. Indeed, as can be noticed from Figure 7(c), both linear and nonlinear methods are not able to fit the data. In particular we point out that often the systems achieve values of nMSE that are larger than 1, implying that for most experiments the number of training examples was not even sufficient to correctly estimate the mean of the distribution of latency values.

Interestingly, a similar behavior is observed for the linear kernel when IPI is used to estimate the latency of neural activity after the second stimulus. On the contrary, the RBF approach is apparently able to learn the dependency between IPI and spike latency, leading to a remarkable decrease of prediction error.

As can be noticed, when varying the stimulus intensity (Figure 7(d)), the nMSE for the latency to 1st and 2nd pulse seems to be comparable with slightly better performances for the Gaussian kernel with respect to the linear one.

## 4. Conclusions

In this study, we took advantage of the peculiar features of the* in vitro* whole brain of guinea pig (namely, great accessibility and controllability of experimental conditions, together with preservation of structure integrity) to deliver repeated paired-pulse stimulations to the LOT, while varying the stimulus features (namely, IPI and stimulus intensity), and record l-ERC responses. Output patterns were first characterized in terms of number of evoked spikes (after both pulses and total), measuring the response strength, and 1st spike latency (after both pulses), measuring the response delay. These data were then used to fit a system (i.e., I/O function), by means of the KRLS method, able to predict l-ERC responses on the basis of LOT stimulation features.

As qualitatively illustrated in Figures 1, 2, and 3, output features are dependent on the input: in fact, gradual increase of either IPI or stimulation intensity causes graded variations of both number of evoked spikes and 1st spike latency, especially when considering the 2nd pulse.

These results are confirmed by mutual information analysis (Figure 5), which revealed how the latency to the 2nd pulse carries the highest information about the input IPI and the number of spikes after the 2nd pulse about the stimulation intensity.

The application of machine learning techniques is effective in modeling the LOT-l-ERC I/O function, especially regarding the prediction of the evoked number of spikes (Figure 6). Prediction accuracies reach very high values with respect to chance levels, thus demonstrating that we were able to reproduce the behavior of the studied circuit by using a statistical modeling approach. Prediction of the time delay between the applied stimulus and the corresponding neural response appears to be more challenging (Figure 7). In particular, only the IPI value and the latency after the 2nd stimulus seem to be significantly correlated, and such correlation is however highly nonlinear (i.e., only the RBF approach allows to actually achieve a remarkable decrease of the error).

The strategy used for LOT stimulation consisted of a paired-pulse protocol at different interpulse intervals and stimulus intensities. These patterns clearly represent an oversimplification of the complex computational tasks carried out in the olfactory cortices to shape an odor percept. To overcome this limitation and convey sensory information through the disrupted synaptic pathway, more naturalistic stimulation patterns based on* in vivo* recordings can be adopted as reported in previous studies [22]. For instance, bursts of stimuli at different frequencies or series of irregular stimuli with random interpulse intervals following different statistics could be used [23]. Moreover, the analysis approach adopted in this work is rather general and independent of the considered input/output features; hence, we believe it might be easily extended to the analysis of more naturalistic stimulation patterns to mimic actual sensory signals.

In conclusion, these results may provide a possible application in the framework of the design and development of innovative cortical prostheses aimed at replacing a damaged part of the brain. Ongoing and future experiments include the application of a mechanical lesion between pPC and l-ERC, thus interrupting the signal transmission from LOT to l-ERC. Similar modeling approaches could be used in principle to derive the optimal local cortical stimulation to be delivered downstream the pPC to restore l-ERC response upon LOT stimulation.
